# On theory and methods for advanced detonation-driven hypervelocity shock tunnels

**DOI:** 10.1093/nsr/nwaa050

**Published:** 2020-04-02

**Authors:** Zonglin Jiang, Jinping Li, Zongmin Hu, Yunfeng Liu, Hongru Yu

**Affiliations:** State Key Laboratory of High Temperature Gas Dynamics, Institute of Mechanics, Chinese Academy of Sciences, Beijing 100190, China; Department of Aerospace Engineering Science, School of Engineering Science, University of Chinese Academy of Sciences, Beijing 100049, China; State Key Laboratory of High Temperature Gas Dynamics, Institute of Mechanics, Chinese Academy of Sciences, Beijing 100190, China; State Key Laboratory of High Temperature Gas Dynamics, Institute of Mechanics, Chinese Academy of Sciences, Beijing 100190, China; Department of Aerospace Engineering Science, School of Engineering Science, University of Chinese Academy of Sciences, Beijing 100049, China; State Key Laboratory of High Temperature Gas Dynamics, Institute of Mechanics, Chinese Academy of Sciences, Beijing 100190, China; Department of Aerospace Engineering Science, School of Engineering Science, University of Chinese Academy of Sciences, Beijing 100049, China; State Key Laboratory of High Temperature Gas Dynamics, Institute of Mechanics, Chinese Academy of Sciences, Beijing 100190, China; Department of Aerospace Engineering Science, School of Engineering Science, University of Chinese Academy of Sciences, Beijing 100049, China

**Keywords:** hypervelocity, shock tunnel, detonation driver, flight condition, tailored condition, test facilities

## Abstract

This study describes theory and methods for developing detonation-driven shock tunnels in hypervelocity test facilities. The primary concept and equations for high-enthalpy shock tunnels are presented first to demonstrate the unique advantage of shock tubes for aerodynamic ground-based testing. Then, the difficulties in simulating flight conditions in hypervelocity shock tunnels are identified, and discussed in detail to address critical issues underlying these difficulties. Theory and methods for developing detonation drivers are proposed, and relevant progress that has advanced the state of the art in large-scale hypersonic test facilities is presented with experimental verifications. Finally, tailored conditions for detonation-driven shock tunnels are described, laying a solid foundation to achieve long test duration. This interface-matching key issue encountered in developing shock tunnels has been investigated for decades, but not solved for detonation drivers in engineering applications.

## INTRODUCTION

Great success has been achieved in aeronautics and astronautics since the first flight powered with an engine was made successfully by the Wright brothers in 1903. Practical supersonic flights were realized with Concorde in 1960, and the space station was also launched successfully. It is believed that we will soon move into the era of hypersonic flight, allowing individuals to travel anywhere on Earth within 2 hours. The cost for space access would become more reliable, routine and affordable if a Multi-Stage-To-Orbit (MSTO) system was realized, featuring capabilities of horizontal takeoff and landing. The impact of hypersonic flight technology on human civilization and modern society is likely to be significant.

In the past two decades, several hypersonic flight tests with different mission targets have been carried out [1]. These were great milestones in aviation and aerospace history; however, in practical terms, hypersonic flight is still only a distant dream, as some critical problems arose from the hypersonic flight tests, which puzzled the design engineers [2,3]. The first is aerodynamic heating, which takes place when hypersonic vehicles fly in the atmosphere. This is induced by both the bow shock wave and the severe viscous friction that transfers kinetic energy to thermal energy, heating the air around the vehicles to thousands of degrees. The relevant heat flux intensity depends on Mach numbers and flight altitudes, and may be strengthened by shock/boundary interaction. Such aerodynamic heating means that thermal-protection systems are needed for hypersonic vehicles. The second problem is related to thermo-aerodynamic processes, and the so-called ‘real gas’ effect. The high temperature of boundary layers around hypersonic vehicles excites air molecule vibrations, and induces oxygen and nitrogen dissociations and even atom ionizations. The hypersonic air flow becomes a special medium undergoing chemical reactions as the flow temperature varies. Consequently, the hypersonic flow becomes a chemically reacting gas flow, accompanied by energy exchanges and heat transfers. This energy exchanges are so large that the hypersonic flow could be dramatically affected in various aspects.

There are at least three critical physical issues that make hypersonic flow different from classic aerodynamics [4–6]. The first is the composition of the chemically reacting gases. Not only are nitrogen and oxygen atoms present, but also nitric oxide. The second issue is the relaxation time, the period when the flow reaches thermodynamic equilibrium state from the beginning of thermo-chemical reactions. Knowing this time scale, one can decide when the gas state becomes equilibrium, and when it is still in a non-equilibrium state. The last issue is the gas property, including the heat conduction parameter, the diffusion and viscosity coefficient, the collision cross section, and chemical reaction rates, etc. Addressing these critical issues would contribute greatly to development of hypersonic vehicles. For example, regarding chemically reacting flows, the stagnation temperature of hypersonic flights at Mach number 12 is only half of the temperature calculated under the ideal gas assumption at the same Mach number. Therefore, the real gas effect is of significant importance not only for physical understanding but also for hypersonic vehicle development [6].

Flight test data show obvious differences between wind tunnel data and computational results. Considering such discrepancies, Bertin pointed out that there is still no ground-based test facility that can duplicate the thermal environment of hypersonic vehicles [3,4]. The ground testing data are limited to flows generated with available hypersonic wind tunnels. The computational results are limited to physical models proposed based on experimental observations, and have nothing to do with computer power and grid mesh sizes. Both experiments and computations involve one critical issue: how to model chemically reacting flows reasonably. The test flow depends on the wind tunnel technology and the physical models in computation depend on the cognition of aero-thermodynamic phenomena through ground-based experiments. Therefore, the primary question is how to obtain reliable experimental data? Developing advanced hypersonic wind tunnels to duplicate the key parameters of hypersonic flight condition has been a challenging research topic for decades [7].

After more than 60 years of research work, hypersonic ground test facilities suitable for exploring aero-thermochemistry still rely on shock tunnels. Many shock tunnels have been built across the world, for example, the Large Energy National Shock tunnels (LENS) in USA, the High-Enthalpy Shock Tunnel (HIEST) in Japan, the High-Enthalpy Shock Tunnel (HEG) in Germany, and the JF-10 high-enthalpy shock tunnel, JF-12 hypersonic flight duplicated shock tunnel and JF-16 hypervelocity expansion tunnel in China [7–9]. Valuable data have been produced with these shock tunnels for both hypersonic gas dynamics and hypersonic vehicle development. Among these facilities, the JF-12 hypersonic flight duplicated shock tunnel (JF-12 or Hyper-dragon I) is the largest in the world and its performance covers Mach numbers from 5 to 9 and flight altitudes from 25 to 50 km [8]. In 2018, another project was launched with support from the National Natural Science Foundation of China, with the objective of building a detonation-driven high-enthalpy hypervelocity shock tunnel (JF-22 or Hyper-dragon II) for covering Mach numbers from 10 to 25 and flight altitudes from 30 to 90 km. In this study, the theory and fundamental equations for detonation-driven hypervelocity shock tunnels are summarized based on the research work on JF-12 and JF-22, and related theory and critical technologies aiming at duplicating hypersonic flight conditions are introduced and discussed in detail to demonstrate the merit and reliability with shock tunnel test verifications.

## SHOCK TUNNELS AND FLIGHT CONDITIONS

The total enthalpy of hypervelocity flows is very high and its stagnation temperature may vary from 1500 K to 10000 K according to flight Mach numbers from 5 to 20. How to generate such a high-enthalpy flow while avoiding tunnel structure damages caused by the thermal load during wind tunnel operation is a critical problem that must be considered in hypersonic tunnel development [7]. To increase flow temperatures, air-heating wind tunnels are widely used all over the world, and test flows with Mach numbers as high as 7 can be generated if the sound speed is simulated to be that at flight altitudes. Technical barriers imposed on this kind of wind tunnel are not only the capacity of its heater power, but also the heat endurance of wind tunnel structure during air-heating processes. Therefore, rapid generation and immediate application of high-temperature reservoir gases could be an ideal way for hypersonic wind tunnels to operate with low thermal loads. From shock wave dynamics, the normal shock wave is well known to be an effective tool for gas compression. For example, if the incident shock Mach number is 5, the compressed gas pressure will be 20–30 bars while the gas temperature can reach 1500 K. Fortunately, it is easy to produce such a shock wave with shock tubes in a laboratory for investigating chemically reacting flows [6].

**Figure 1.**
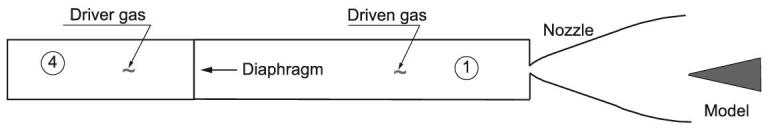
Schematic diagram of a shock tunnel.

The shock tunnel is an extension of shock tubes, and its primary concept is shown in Fig. 1. A simple shock tunnel is realized by connecting a nozzle to the end of the driven section. The nozzle is initially separated from the driven section by a diaphragm. When a shock tunnel works, the incident shock wave is reflected at the end of the driven section, resulting in a column of stationary gases with total pressure and stagnation temperature the same as those of flight conditions. This column of stationary gases acts as a high pressure gas reservoir for wind tunnels.

**Figure 2.**
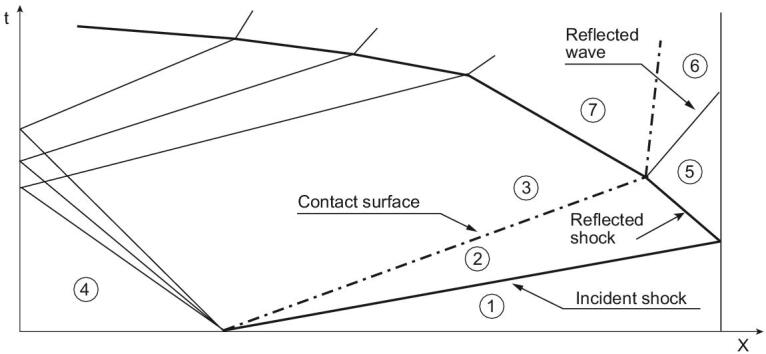
Wave diagram of a shock tunnel during its operation.

During shock tunnel operation, region 5 is developed behind the reflected shock wave, and the gas reservoir is generated with the required total pressure and stagnation temperature. After nozzle diaphragm rapture, the high pressure gas rushes into the nozzle, and experiences an acceleration and expansion process. The test flow develops at a required Mach number corresponding to the nozzle expansion ratio. Figure 2 presents the x−t diagram of the wave system in the whole shock tunnel during its operation. The relation between the incident shock Mach number and thermodynamic parameters in region 5 can be expressed by the following equations:

(1) \begin{eqnarray*} \frac{{{p_5}}}{{{p_1}}} = \frac{{\left[ {2{\gamma _1}M_s^2 - \left( {{\gamma _1} - 1} \right)} \right]\left[ {\left( {3{\gamma _1} - 1} \right)M_s^2 - 2\left( {{\gamma _1} - 1} \right)} \right]}}{{\left( {{\gamma _1} + 1} \right)\left[ {\left( {{\gamma _1} - 1} \right)M_s^2 + 2} \right]}},\hphantom{00000000000000000000000000000000000} \end{eqnarray*}

(2) \begin{eqnarray*} \frac{{{T_5}}}{{{T_1}}} = \frac{{\left[ {2\left( {{\gamma _1} - 1} \right)M_s^2 - \left( {{\gamma _1} - 3} \right)} \right]\left[ {\left( {3{\gamma _1} - 1} \right)M_s^2 - 2\left( {{\gamma _1} - 1} \right)} \right]}}{{{{\left( {{\gamma _1} + 1} \right)}^2}M_s^2}},\hphantom{00000000000000000000000000000}\,\, \end{eqnarray*}

(3) \begin{eqnarray*} \frac{{{\rho _5}}}{{{\rho _1}}} = \frac{{\left( {{\gamma _1} + 1} \right)M_s^2\left[ {2{\gamma _1}M_s^2 + 1 - {\gamma _1}} \right]}}{{\left[ {2\left( {{\gamma _1} - 1} \right)M_s^2 - \left( {{\gamma _1} - 3} \right)} \right]\left[ {\left( {{\gamma _1} - 1} \right)M_s^2 + 2} \right]}}.\hphantom{00000000000000000000000000000000000000\,} \end{eqnarray*}

For a shock tunnel designed to duplicate the required flight conditions, *p*_5_ and T_5_ in region 5 can be determined from the flight conditions identified by Mach number and flight altitude. Once the required gas state in region 5 is obtained in the nozzle reservoir, the actual flight condition can be reproduced with the nozzle designed properly by assuming a reversible expansion process. The flight conditions include the flight speed, the static pressure and the static temperature. The latter two parameters are related by flight altitudes.

For shock tunnel calibration, the required Mach number, Ms, of the incident shock wave is calculated from Eq. (2) by assuming the laboratory temperature *T*_1_, once the stagnation temperature and total pressure (*T_5_*, *p_5_*) are obtained from flight conditions. Applying both Ms and *p*_5_, the pressure *p*_1_ in the driven gas can be calculated from Eq. (1). Knowing Ms and *p*_1_, the driver pressure *p*_4_ can be obtained using shock tube equations from aerodynamic textbooks. In this way, shock tunnel operation conditions are defined, and then can be calibrated according to the conditions.

If the incident shock is strong, the specific heat ratio in regions 1 and 2 is not the same because of high post-shock temperatures, i.e., ${\gamma _1} \ne {\gamma _2}$. Consequently, the specific heat at constant pressure, ${C_p}$, can be calculated with the following equations by assuming the gases across the shock wave to be thermally perfect

(4) \begin{equation*}\left\{ \begin{array}{@{}l@{}} {C_{p1}} = \frac{{{\gamma _1}}}{{{\gamma _1} - 1}}{R_1}\quad\left( {{T_1} < {T_C}} \right)\\{C_{p2}} = \frac{{{\gamma _2}}}{{{\gamma _2} - 1}}{R_2}\quad\left( {{T_2} \ge {T_C}} \right) \end{array} \right.,\end{equation*}

where, ${T_c}$ is a characteristic temperature and $\gamma $ changes when $T$ arises over this characteristic temperature. The enthalpy can be derived from ${C_p}$ for a thermally perfect gas, e.g.,

(5) \begin{equation*} {h_1} = \int_{0}^{{{T_1}}}{{{C_{p1}}}}{\rm{d}}T = \frac{{{\gamma _1}}}{{{\gamma _1} - 1}}{R_1}{T_1}\quad \left( {{T_1} < {T_c}} \right), \end{equation*}

(6) \begin{equation*} {h_2} = \int_{0}^{{{T_c}}}{{{C_{p1}}}}{\rm{d}}T + \int_{{{T_c}}}^{{{T_2}}}{{{C_{p2}}}}{\rm{d}}{T_2}\quad \left( {{T_2} \ge T_c} \right), \end{equation*}

or

(7) \begin{equation*}{h_2} = \frac{{{\gamma _2}}}{{{\gamma _2} \!-\! 1}}{R_2}{T_2} - \left( {\frac{{{\gamma _2}}}{{{\gamma _2} \!-\! 1}}{R_2} - \frac{{{\gamma _1}}}{{{\gamma _1} \!-\! 1}}{R_1}} \right){T_c},\end{equation*}

where we introduce a characteristic enthalpy, $\Delta {h_c}$, to represent the second term on the right hand of Eq. (7), i.e., $\Delta {h_c} = ( {\frac{{{\gamma _2}}}{{{\gamma _2} - 1}}{R_2} - \frac{{{\gamma _1}}}{{{\gamma _1} - 1}}{R_1}} ){T_c}$.

**Figure 3.**
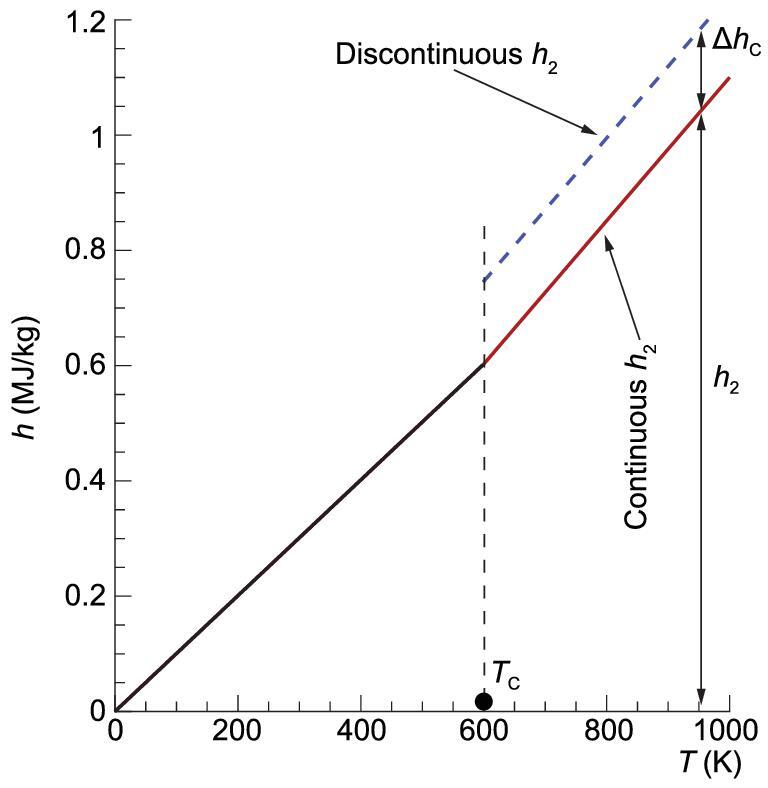
Enthalpy models with γ heterogeneity induced by high-temperature (real-gas) effect.

The enthalpy variation with temperature is schematically depicted in Fig. 3 for imperfect air, where the characteristic temperature ${T_{c\ }}$is supposed to be 600 K. In the figure, labels ‘continuous *h*_2_’ and ‘discontinuous *h*_2_’ denote the enthalpy formulation with and without the term $\Delta {h_c}$, respectively. It can be seen from the curve that $\Delta {h_c}$ does not vary with temperature, therefore, $\Delta {h_c} \ll {h_2}$ and it can be neglected when ${T_2} \gg {T_c}$ in the cases of very strong shock waves. However, for a moderate shock wave, $\Delta {h_c}$ becomes comparable to ${h_2}$ and should not be ignored. Complement equations will be presented in the following paragraph.

The conservation laws for mass, moment and energy across a strong normal shock wave can be written as

(8) \begin{equation*} {\rho _1}{u_1} = {\rho _2}{u_2}, \end{equation*}

(9) \begin{equation*} {\rho _1}{u}_1^2 + {p_1} = {\rho _2}{u}_2^2 + {p_2}, \end{equation*}

(10) \begin{equation*} \hphantom{00000}\frac{1}{2}{u}_1^2 + {h_1} = \frac{1}{2}{u}_2^2 + {h_2}.\end{equation*}

Substituting Eqs (5), (7) and the equation of state, $p = \rho RT$, into Eq. (10), we have

(11) \begin{equation*}\frac{1}{2}{u}_1^2 + \frac{{{\gamma _1}}}{{{\gamma _1} - 1}}\frac{{{p_1}}}{{{\rho _1}}} = \frac{1}{2}{u}_2^2 + \frac{{{\gamma _2}}}{{{\gamma _2} - 1}}\frac{{{p_2}}}{{{\rho _2}}} - \Delta {h_c}.\end{equation*}

From Eqs (8) and (9) we can get

(12) \begin{equation*}{u}_1^2 = \frac{{{\rho _2}}}{{{\rho _1}}}\frac{{\left( {{p_2} - {p_1}} \right)}}{{\left( {{\rho _2} - {\rho _1}} \right)}},\end{equation*}

(13) \begin{equation*}{u}_2^2 = \frac{{{\rho _1}}}{{{\rho _2}}}\frac{{\left( {{p_2} - {p_1}} \right)}}{{\left( {{\rho _2} - {\rho _1}} \right)}}.\end{equation*}

Substituting Eqs (12) and (13) into (11), we have the Rankine-Hugoniot (RH) relation in a general form, which can be written as

(14) \begin{equation*} \frac{p_2}{p_1} = \frac{{({\frac{{{\gamma _1} + 1}}{{{\gamma _1} - 1}} + 2\Delta {{\tilde{h}}_c}})\frac{{{\rho _2}}}{{{\rho _1}}} - 1}}{{\frac{{{\gamma _2} + 1}}{{{\gamma _2} - 1}} - \frac{\rho _2}{\rho _1}}}, \end{equation*}

where $\Delta {\tilde{h}_c}$ represents the dimensionless enthalpy difference and reads

(15) \begin{equation*} \Delta {\tilde{h}_c} = \Delta {h_c}\frac{{{\rho _1}}}{{{p_1}}} = \left( {\frac{{{\gamma _2}}}{{{\gamma _2} - 1}}\frac{{{R_2}}}{{{R_1}}} - \frac{{{\gamma _1}}}{{{\gamma _1} - 1}}} \right)\frac{{{T_c}}}{{{T_1}}}. \end{equation*}

It should be noted that $\Delta {\tilde{h}_c}$ is a model parameter involving the characteristic temperature ratio, ${{{T_c}} /{T_1}}$. If one wants to consider the real gas effect to get a more accurate prediction of the shock tube problem, the above equations can provide a base for modification of the traditional shock relations. For hypersonic flows in the presence of strong shock waves where the high-temperature effects are critical, Eq. (14) shall be used. The following relation holds ahead of the shock wave,

(16) \begin{equation*}{u}_1^2 = {M}_1^2{a}_1^2 = M_1^2{\gamma _1}\frac{{{p_1}}}{{{\rho _1}}}.\end{equation*}

Substituting Eq. (16) into Eq. (12) yields the relation among the flow Mach number ahead of the shock wave and the pressure and density ratios

(17) \begin{equation*}\frac{{{p_2}}}{{{p_1}}} = \frac{{\left( {{\gamma _1}M_1^2 + 1} \right)\frac{{{\rho _2}}}{{{\rho _1}}} - {\gamma _1}M_1^2}}{{\frac{{{\rho _2}}}{{{\rho _1}}}}}.\end{equation*}

Combining Eqs (14) and (17), we obtain a quadratic equation about the unknown density ratio, ${{{\rho _2}} / {\rho _1}}$, across the normal shock wave,

(18) \begin{eqnarray*} && \left[ {{\gamma _1}M_1^2 + \frac{{2{\gamma _1}}}{{{\gamma _1} - 1}} + 2\Delta {{\tilde{h}}_c}} \right]{\left( {\frac{{{\rho _2}}}{{{\rho _1}}}} \right)^2} \nonumber\\ &&\quad -\, \left[ {\frac{{2{\gamma _2}}}{{{\gamma _2} - 1}}\left( {{\gamma _1}M_1^2 + 1} \right)} \right]\frac{{{\rho _2}}}{{{\rho _1}}} \nonumber\\ &&\quad +\, \left[ {\frac{{{\gamma _2} + 1}}{{{\gamma _2} - 1}}{\gamma _1}M_1^2} \right] = 0. \end{eqnarray*}

Thus, from Eq. (18) we get the shock relation in a general form

(19) \begin{eqnarray*} \frac{{{\rho _2}}}{{{\rho _1}}} = \frac{{\frac{{{\gamma _2}}}{{{\gamma _2} - 1}}\left( {{\gamma _1}M_1^2 + 1} \right) + \frac{{\sqrt {{{\left( {{\gamma _1}M_1^2} \right)}^2} - 2\left[ {\frac{{\gamma _2^2 - {\gamma _1}}}{{{\gamma _1} - 1}} + \left( {\gamma _2^2 - 1} \right)\Delta {{\tilde{h}}_c}} \right]{\gamma _1}M_1^2 + \gamma _2^2} }}{{{\gamma _2} - 1}}}}{{{\gamma _1}M_1^2 + \frac{{2{\gamma _1}}}{{{\gamma _1} - 1}} + 2\Delta {{\tilde{h}}_c}}}.\hphantom{000000000000000000000000000000\,}\end{eqnarray*}

Other quantities across strong shock waves can be obtained accordingly. With the strong shock relations, we can solve the shock tube problem when the high-temperature effect is considered. The theoretical analysis on shock tunnel operations in this section demonstrates that hypersonic flight conditions can be duplicated if the shock tunnel driver is powerful enough. For ground-based hypersonic test facilities, developing powerful drivers for shock tunnels has been a challenging topic within the shock tube community for decades.

## THEORY AND METHODS FOR DETONATION DRIVERS

Theoretical analysis on shock tunnels indicates that it is feasible to duplicate flight conditions in the laboratory. However, there are still two important issues to consider for ground-based hypersonic tests. The first is non-equilibrium process simulation and the second is interface-matching conditions. These two issues will be discussed in the following text. Regarding the reentry problem of a spacecraft at a speed of 6000 m/s, dissociation of a nitrogen molecule is induced by bond breakdown of two nitrogen atoms because of strong vibration. The relaxation time at atmospheric pressure is about 1/1000 s at temperature around 2000 K, and about 10^−7^ s at ∼7000 K [6]. The equilibrium length is 6 m for the former relaxation time, and 0.06 cm for the latter. This indicates that if the fuselage is 6 m long, the hypersonic flow outside the fuselage cannot reach its equilibrium states. This is because the post shock temperature is very high behind the normal part of the bow shock, but is lower and lower behind the oblique part as the air flow is further and further away from the normal shock. Therefore, it is necessary for a reliable ground test facility to produce a sufficiently large test flow region to accommodate a test model that is large enough to minimize non-equilibrium effects on hypersonic vehicles [6]. It is a key issue for shock tunnels to generate not only the pure air flow with the right temperature to simulate the correct chemistry, but also the large test flow field for chemical reaction processes to complete. With regard to the size of test flow fields, a nozzle diameter from 2.5 to 3 m is necessary for most hypersonic vehicles in flight tests over the world. With such large nozzles, a

full-scale test model could be accommodated in test flows. Actually, the predominant physical process in hypersonic flows is high-temperature-induced thermochemical reactions, and the characteristic reaction scale does not change when a vehicle is scaled to a small test model. It means that the model-scaling theory widely applied in traditional supersonic wind tunnel experiments is no longer applicable for hypersonic experiments.

In hypersonic research area, there is binary scaling for non-equilibrium processes involving two-body molecular collisions [4]. This binary scaling parameter has been demonstrated for the blunt-body stagnation shock layer where the non-equilibrium process is dominated by the two-body dissociation reactions rather than the three-body recombination reactions [10]. However, the binary scaling is not applicable for air-breathing hypersonic vehicles such as X-43a and trans-atmospheric vehicles such as X-37b. The X-43a has an integrated configuration involving a scramjet engine in which the supersonic combustion is governed by much more complex chemical reactions. For X-37b, the flow-field right behind its bow shock is dominated by the two-body dissociation, but in its boundary layer, the recombination reaction takes place as the flow temperature decreases downstream from the stagnation region. Furthermore, for the hypersonic boundary layer transition, the surface roughness and catalytic reactions are also not scalable. Hypersonic flight tests in recent years showed obvious discrepancies from wind tunnel experiments, but agree well with full-scale model tests of the Hyper-dragon I. This is a challenge not only for hypersonic ground test facilities but also for scaling law researches for hypersonic flows. However, if we could construct hypervelocity wind tunnels to accommodate full-scale models of the present flight-testing hypersonic vehicles, and then draw the correct correlation between flight test data and wind tunnel experimental results for scaled models, the work will be of significant importance for developing future hypersonic vehicles which may have much larger dimensions.

In summary, for reliable hypersonic ground tests, there are four requirements that must be considered carefully in wind tunnel development: (1) the test gas, without any additions, must be the pure air to accurately simulate chemical reaction mechanisms; (2) the stagnation temperature and total pressure must be achieved to excite the correct chemical reactions; (3) the scale of the test model must be large enough to ensure that chemical reactions occur at the correct reaction rates at the right location on the test models; (4) a sufficiently long test time is necessary for aerodynamic forces and supersonic combustion tests. The fourth requirement is important for the test flow to reach stable combustion and to improve experimental data accuracy of aerodynamic forces and moments. Meeting these four requirements at the same time would result in duplication of flight conditions in test facilities, but this has been a challenge for developing hypersonic test facilities for decades.

The first difficulty arising from the four requirements discussed above is the power requirement for shock tunnel drivers. If one wants to produce the flight condition at 40 km altitude for Mach number 15, the output power of shock tunnels needs to be ∼1056 MW to generate a nozzle flow being 2.5 m in diameter. The output power is ∼2054 MW at altitude of 35 km. The input power for shock tunnels is much higher than the output power. Such power demand is necessary not only to achieve high-enthalpy gas states, but also to compress a large amount of air mass to such a thermal state for a long test duration.

We discuss a free-piston driver first as the driver is widely applied to high-enthalpy shock tunnels over the world. This class of shock tunnels have been used to generate most of the valuable data for the high-enthalpy flow research [7]. However, the free-piston driven shock tunnel technique has three limitations in meeting the four requirements mentioned above. The first limitation is the power requirement. The total energy is ∼45 MJ for a 1000 kg mass piston moving at about 300 m/s. Although the output power is ∼450 MW for the 100 ms test duration, this value is still much lower than the requirements because the efficiency of the energy transfer from the piston to the test gas is not considered. For flights at low altitudes the requirement for the total energy carried by the free piston could be even bigger. Actually, the piston mass and speed in this case are the maximum values that were reached in the HEIST operation. Therefore, the free-piston driver works well for shock tunnels to produce high-enthalpy flows with a short test duration, but has its limitation in meeting the need from large-scale hypersonic test facilities which would operate for long test duration.

The second limitation is that the test duration is not limited only by the output power requirement. For the free-piston driven shock tunnel, the pressure in front of the moving piston will fall rapidly once the diaphragm ruptures and the phenomenon results in rapid decay of the incident shock wave. The tuned operation mode can be applied to improve on the incident shock decay and the test duration of the free-piston driven shock tunnels could be extended, but is still measured in milliseconds. For example, the test duration for HEIST is between 2 and 5 ms. The last is the moving part of the free piston drivers. Heavy pistons moving at high speeds lead to technical difficulties in operation for piston launching and stopping. The heavier the piston, the more difficult is the shock tunnel operation.

**Figure 4.**
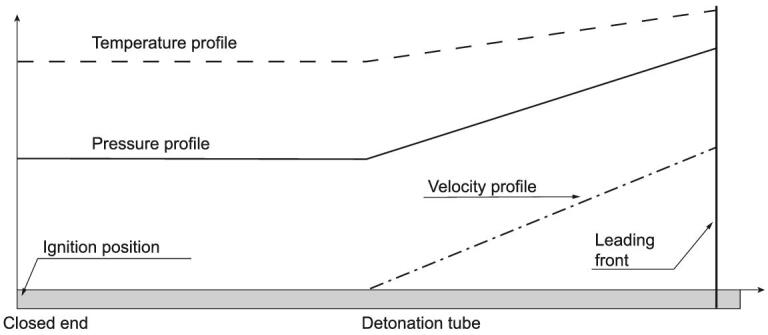
Schematic diagram of the distribution of detonation wave properties along the length of a detonation tube and calculated with the C-J theory.

We considered the detonation driver as a power supply for shock tunnels as detonation phenomena are well known for a powerful energy release rate. To explain the detonation driver and its working modes, we take a circular steel tube with one end closed as a detonation tube, and fill it with detonable gas mixtures. A detonation wave is initiated at its closed end and propagates towards the other end. According to the Taylor expansion wave theory, the pressure and velocity distributions along the detonation tube are shown schematically in Fig. 4. As a result of the stationary boundary condition at its closed end, an expansion wave system is developed behind the detonation front through which the gas flow velocity gradually decreases to a stationary state. The column length of the detonated-gas at rest, which still has very high temperature and pressure, is about half of the propagation distance of the detonation front. Bird made the first attempt to use the super-high power of detonations for high-enthalpy shock tunnels [11], and the key point for developing detonation drivers is how to generate, control and apply detonations safely to obtain stable incident shock waves in driven sections. Yu proposed a concept by appending a damping section to the open end of a detonation tube for accommodating the huge energy carried by the detonation front to ensure the shock tunnel operation safety. A driven section is also appended to the closed end of the detonation tube to get a stable incident shock wave. This concept of the backward detonation-driven shock tunnel with a damping section was applied successfully to the TH2-D high-enthalpy shock tunnel in the Aachen University of Technology [12], and then to the JF-10 shock tunnel in the Institute of Mechanics, CAS [13].

**Figure 5.**
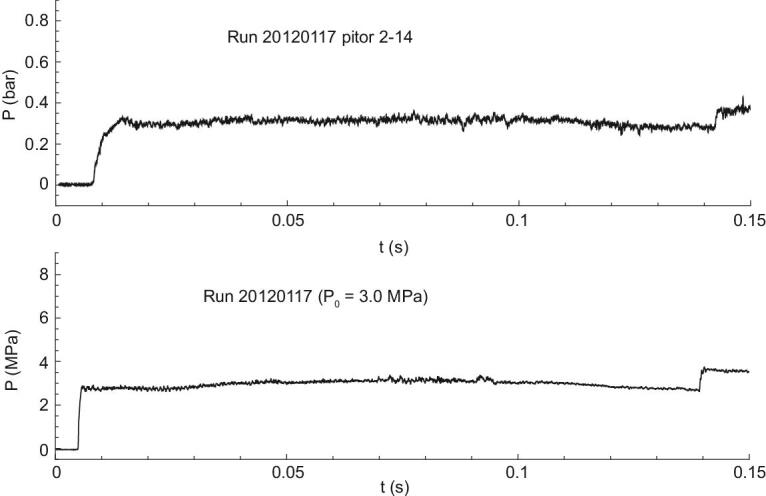
Reservoir pressure *p*_5_ (low) and pitot pressure (upper) histories from performance calibration of Hyper-dragon I.

Extensive study on the backward detonation driver was carried out during development of the Hyper-dragon I, and two important technologies were proposed to obtain a perfect incident shock wave [8,14]. The first is direct detonation initiation, achieved by a special igniter with multi-stage expanding diameter. With the igniter, multi-stage amplifications of the released energy are realized to accumulate a large amount of hot gases to directly initiate a detonation wave. The other technology is a pre-formed diaphragm with critical thickness. Such a diaphragm can reduce significantly the energy loss during its breakage and avoid fragments from the diaphragm. Figure 5 shows the *p*_5_ curve and the pitot pressure distributions from calibrations of Hyper-dragon I. The sharp jump and the uniform distribution indicate excellent performances of the backward detonation driver and demonstrate well the role of the two technologies mentioned above. For the Hyper-dragon I, the maximum stagnation temperature of test flows is ∼4000 K and the total pressure that can be achieved is 6 MPa. The test duration of Hyper-dragon I is ∼130 ms and is achieved with 200 m length of the driven and driver sections.

By looking back to Fig. 4, it can be seen that the detonation front has much higher energy than the stationary gases behind it. This is because the detonated gas pressure behind the detonation front is much higher than the stationary part and the gas velocity is not zero. Attaching a driven section to the open end of a detonation tube makes a forward detonation driver. Theoretically speaking, the driving power of the forward detonation driver is about five times higher than the backward detonation driver at the same initial conditions when measuring with the C-J detonation parameters. However, the Taylor expansion wave leads to a gradual attenuation of the incident shock wave, and this indicates that the forward detonation driver is not acceptable for practical applications to shock tunnels.

**Figure 6.**
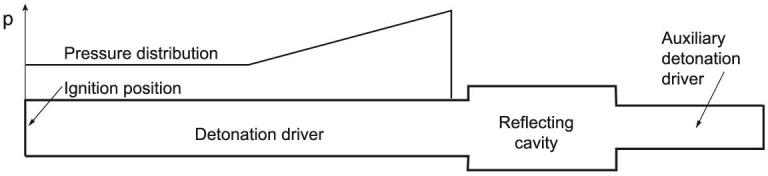
Schematic diagram of the FDC driver with pressure distribution.

To solve the above mentioned attenuation problem, Jiang *et al*. proposed the Forward Detonation Cavity (FDC) driver, as schematically shown in Fig. 6, based on the shock reflection concept [13]. The FDC driver consists of three parts: the detonation driver, the reflecting cavity and the auxiliary detonation driver. When the detonation wave with a pressure distribution as shown in Fig. 6 passes through the reflecting cavity, its middle part propagates directly into the auxiliary detonation driver while the circumferential part is reflected back from the end wall of the cavity and forms a reflected shock wave that is travelling upstream. The incoming flow passes through the reflected shock wave, and the shock interaction results in its pressure and temperature increase. Considering the shock system developed in the FDC driver, it is understood that the stronger upstream-travelling shock wave and the weaker downstream-travelling shock wave from shock interaction are favorable for enhancing the FDC driver performance. This is also the principle for optimization of the FDC driver.

**Figure 7.**
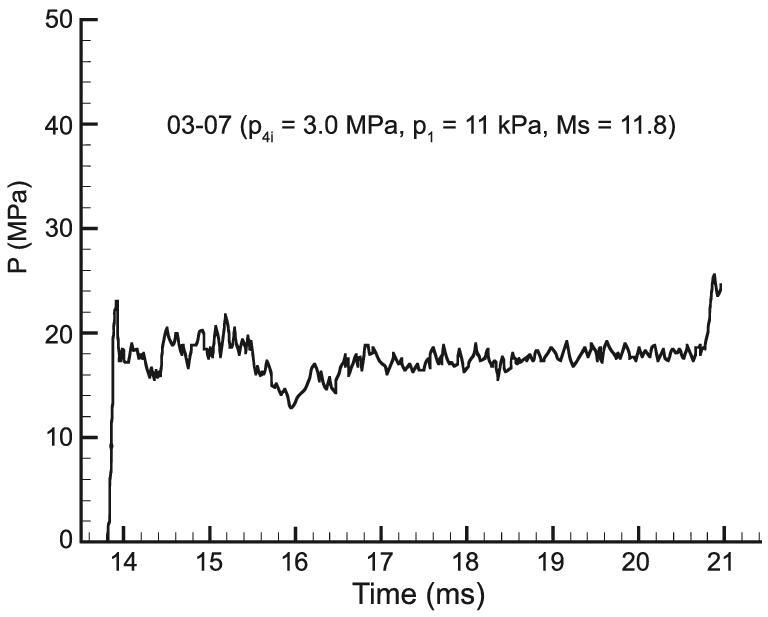
*p*_5_ of the JF-10 detonation-driven high-enthalpy shock tunnel.

The FDC driver was successfully applied to the JF-10 detonation-driven high-enthalpy shock tunnel at the Institute of Mechanics, CAS, and the shock tunnel was used to generate high-enthalpy flows with a total temperature up to 7000 K. One of the *p*_5_ curves from the JF-10 shock tunnel performance tests is presented in Fig. 7. The uniformity of the pressure distribution shows that the FDC driver successfully eliminates the incident shock attenuation caused by the Taylor expansion wave, and fluctuations around the average pressure damp rapidly in the driven section. The 6 ms test time demonstrated excellent performance of the JF-10 shock tunnel, which is only about 25 m long [13].

**Figure 8.**
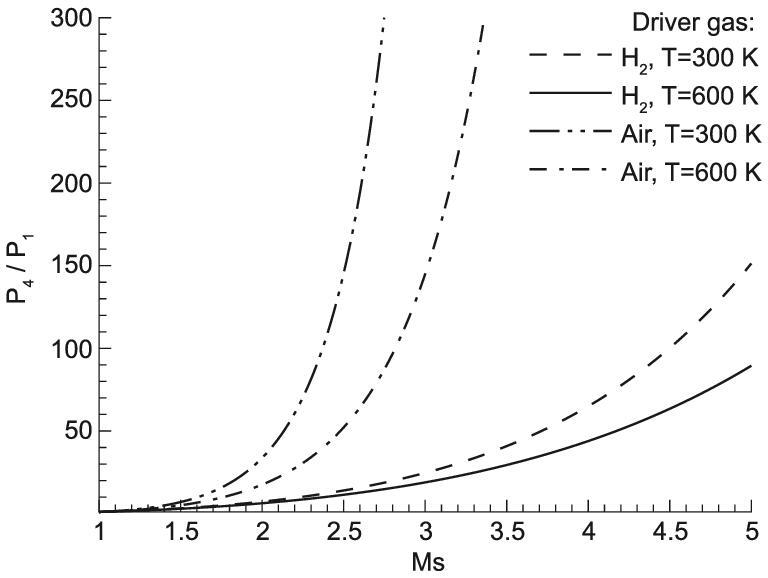
Driving pressure ratio versus incident shock Mach number for driving gases at different temperature.

The strength of the incident shock waves in the driven section depends on two issues. The first is the composition of the driver gas. From Eq. (14) one can find that the incident shock Mach number is determined for driven gases once the driver gas composition and its thermodynamic state in region 4 are given. Figure 8 shows the relation between the pressure ratio *p*_4_/*p*_1_ and the incident shock Mach number for different gases. If air at room temperature is used as the driver gas, the pressure ratio *p*_4_/*p*_1_will increase dramatically when the required Mach number of the incident shock wave is >2. This means that increasing the pressure ratio is an effective method to increase the incident shock Mach number, but high Mach numbers >2.5 are not practical in engineering because the required pressure ratio will approach infinity rapidly (Fig. 8). The second issue is the driver gas temperature. Heating air to 600 K can increase its sound speed, which will increase the incident Mach number, but the practical Mach number of the heated air is not >3. Light gases have high sound speeds that can be also increased if heated. From Fig. 8, it can be seen that the incident shock Mach number can be as high as 5 using hydrogen as a driver gas. Heating hydrogen to 600 K could help to get the incident shock Mach number >5. However, heating and discharging large amounts of hydrogen pose a serious safety problem for shock tunnel operation. Heated helium can be used to replace hydrogen as a driver gas to solve the problem arising from heated hydrogen, and was once considered as one of the JF-12 operation modes. To generate the 2.5 m diameter test flow of 5 MPa total pressure for Mach 9 at 40-km altitude, the driver gas mass required is about 187 kg helium for the light-gas-heated driver, and 7 kg hydrogen for the hydrogen/oxygen detonation driver. This is a limitation to the helium-heated driver becoming an affordable technology for large-scale high-enthalpy shock tunnels.

For detonation drivers, for example, the product of hydrogen-oxygen detonations is water vapor with a low specific heat ratio, but with a temperature as high as 3000 K. This temperature is so high that it could be very difficult to achieve with current gas-heating technologies. This is key to the detonation driver being used to generate incident shock waves for high Mach numbers. In addition, the product composition and the detonation temperature can be adjusted as required by diluting with inert gases to meet the interface-matching conditions. This advantage is of significant importance for high-enthalpy shock tunnels to achieve stable reservoir states for long test duration.

## INTERFACE-MATCHING CONDITIONS FOR DETONATION-DRIVEN SHOCK TUNNELS

The test duration of shock tunnels is a key performance parameter, and is usually measured in milliseconds. For the large free-piston-driven HIEST, the test duration is about 2–5 ms. Holden commented that traditional shock tunnels are suitable only for measurements of the cold wall heat flux. An air-breathing propulsion, rocket-plume interaction, steady aerodynamic force measurement can be made only if the test duration is prolonged effectively. The Hyper-dragon I can provide 100 ms test duration, and extensive work has been carried out to extend LENS’ test time [15].

The test duration is closely related to wave propagations during shock tunnel operation. There are two processes that play an important role. One is the shock wave reflected from the damping section and propagating towards the nozzle throat. The total length of driver and driven sections of the Hyper-dragon I is about 200 m, to obtain about 100 ms test duration. The second process is interaction of the reflected incident shock wave with the interface separating the driver gas from the driven gas. No matter whether shock waves or expansion waves are reflected back from the interface, the gas state in the shock tunnel reservoir will be changed, and the effective test duration is shortened consequently. To obtain a long test duration, avoiding any wave reflection from the interface is the best choice for shock tunnel operation. Such tuned interface is called the ‘interface-matching condition’, also known as the ‘tailored operation condition’. The test duration can be increased by several orders of magnitude if the shock tunnel is operated in this condition.

For the interface-matching condition, the acoustic resistance for the gases at the two sides of the interface must be identical so that the shock wave can pass freely without any reflection. Under such conditions it holds, ${p_5} = {p_7}$, ${u_5} = {u_7} \approx 0$. As there is no pressure gradient across the interface, we can get${p_2} = {p_3}$, ${\rm{\ }}{u_2} = {u_3}$. The relation for the reflected shock wave that is a left-running shock in region 2 is given by

(20) \begin{eqnarray*} \frac{{{u_2}}}{{{a_2}}} =\nonumber\\ \left( {\frac{{{p_5}}}{{{p_2}}} - 1} \right)\Bigg/\sqrt {\frac{{{\gamma _2}\left( {{\gamma _2} - 1} \right)}}{2}\left[ {\frac{{\left( {{\gamma _2} + 1} \right){p_5}}}{{\left( {{\gamma _2} - 1} \right){p_2}}} + 1} \right]}.\nonumber\\ \end{eqnarray*}

The transmitted shock wave is a left-running wave in region 3, and

(21) \begin{eqnarray*} \frac{{{u_3}}}{{{a_3}}} \nonumber\\ = \left( {\frac{{{p_7}}}{{{p_3}}} - 1} \right)\!\!\Bigg/\!\!\sqrt {\frac{{{\gamma _3}\left( {{\gamma _3} - 1} \right)}}{2}\left[ {\frac{{\left( {{\gamma _3} + 1} \right){p_7}}}{{\left( {{\gamma _3} - 1} \right){p_3}}} \!+\! 1} \right]} \nonumber\\ = \left( {\frac{{{p_5}}}{{{p_2}}} - 1} \right)\!\!\Bigg/\!\!\sqrt {\frac{{{\gamma _3}\left( {{\gamma _3} - 1} \right)}}{2}\left[ {\frac{{\left( {{\gamma _3} + 1} \right){p_5}}}{{\left( {{\gamma _3} - 1} \right){p_2}}} \!+\! 1} \right]}.\nonumber\\ \end{eqnarray*}

Dividing Eq. (20) by Eq. (21), and setting ${\gamma _2} = {\gamma _1}$ and ${\gamma _3} = {\gamma _4}$, we can obtain

(22) \begin{equation*} \frac{{{a_2}}}{{{a_3}}} = \frac{{{\gamma _1}}}{{{\gamma _4}}} \left[ {\frac{{1 + \frac{{\left( {{\gamma _1} + 1} \right)}}{{2{\gamma _1}}}\left( {\frac{{{p_5}}}{{{p_2}}} - 1} \right)}}{{1 + \frac{{\left( {{\gamma _4} + 1} \right)}}{{2{\gamma _4}}}\left( {\frac{{{p_5}}}{{{p_2}}} - 1} \right)}}} \right]. \end{equation*}

Substituting the expansion relation between regions 4 and 3, we achieve the interface-matching condition for the driver/driven gas interface and it reads as

(23) \begin{eqnarray*} \frac{{{a_4}}}{{{a_1}}} = \frac{2}{{{\gamma _1} \!+\! 1}}\left( {{M_s} - \frac{1}{{{M_s}}}} \right) \bigg\{\left[ {\frac{{({{\gamma _4} - 1})M_s^2 + 2}}{{2{\gamma _1} ({M_s^2 - 1})}}} \right] \nonumber\\ \times {\left[ {\gamma _4^2 + \frac{{{\gamma _1}{\gamma _4} ({{\gamma _4} + 1}) ({M_s^2 - 1})}}{{({{\gamma _1} - 1}) M_s^2 + 2}}} \right]}^{0.5} + \frac{{{\gamma _4} - 1}}{2} \bigg\}. \nonumber\\ \end{eqnarray*}

From the above equation, it can be seen that the tailored Mach number Ms is a function of the sound speed ratio a_4_/a_1_, and the specific heat ratio ${{\rm{\gamma }}_1}$ and ${{\rm{\gamma }}_4}$ of the driver and the driven gases, respectively. As long as the composition and the initial temperature of the driver and the driven gases are known, a_4_/a_1_ can be determined. And then the tailored Mach number can be obtained by solving Eq. (23). This equation works for the backward-running detonation driver as the incident shock wave is stable, and it does also for the FDC driver when it operates in its optimized configuration design condition. It is important to emphasize that the tailored interface is effective only for a given Mach number of the stable incident shocks, the so-called ‘tailored Mach number’. The test gas is air usually at room temperature, therefore, only the driver gas and its state can be adjusted to meet with Eq. (23). The tailored Mach number can be determined when the driver gas and its thermal state are given, and the total enthalpy of the test gas can also be determined with the Mach number.

**Figure 9.**
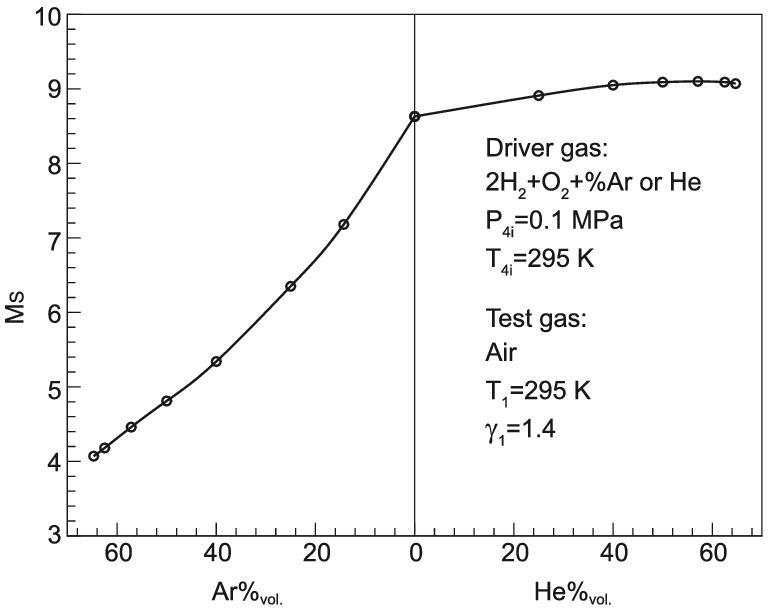
Incident shock Mach number for the stoichiometric hydrogen/oxygen mixture at tailored conditions versus the concentration of helium or argon dilution.

For detonation drivers, the tailored Mach number can be obtained by adjusting the driver gas composition so that the test flow with required total enthalpies can be generated. It is important to point out that the detonated gas must be used for calculation rather than the initial detonable gas mixtures. Results calculated with Eq. (23) are presented in Fig. 9 for detonation-driven shock tunnels [8,16]. The figure shows that the tailored Mach number increases with increasing helium concentration of the initial driver gas, and decreases with increase of the argon concentration fraction.

For the tailored Mach number, similar effects can be achieved by adding hydrogen or nitrogen to the driver gas. Decreasing the argon concentration will increase not only the sound speed of detonated gases, but also its temperature as the chemical energy release is also increased. The dual-parameter effect on the sound speed for the detonation driver can be used to help match the interface condition. For a stoichiometric hydrogen/oxygen mixture, it is also effective to increase the tailored Mach number by adding helium because dilution with helium can make the detonated gases lighter. However, the detonation temperature will decrease because of the low chemical energy release. For the detonation driver operated with a hydrogen/oxygen mixture diluted with helium, a tailored Mach number as high as 9 can be achieved. This Mach number can be applied to generate high-enthalpy flows of total temperatures up to 8000 K. The expansion tube has to be accepted if higher enthalpy flow is needed. Jiang *et al*. reported progress on the JF-16 hypervelocity expansion tunnel in which a hypervelocity flow at 10 km/s can be achieved [9].

## CONCLUSION

The theory and methods are proposed for detonation-driven hypervelocity shock tunnels, and three aspects are discussed for developing such advanced hypersonic test facilities. The first is the special feature of shock tunnels with which the stagnation temperature and the total pressure of test flows can be simulated selectively to generate hypersonic flows with a required velocity but at different altitudes. The second regards high-power detonation drivers that are demonstrated to meet four demands from large-scale high-enthalpy hypersonic testing. Two kinds of detonation drivers are developed, the backward detonation driver for long test duration and the FDC driver for high-enthalpy flows. The third aspect deals with the interface-matching conditions, which are good for improving test flow quality and keeping test time as long as possible. Two detonation drivers can be operated under the interface-matching conditions to yield incident Mach numbers as high as 9. Based on the proposed theory and methods, it is possible to develop large-scale hypersonic test facilities for thermal-aerodynamic research on chemically reacting gas flows.
